# Socioeconomic status in childhood and obesity in adults: a population-based study

**DOI:** 10.11606/S1518-8787.2018052000123

**Published:** 2018-02-07

**Authors:** Katia Jakovljevic Pudla Wagner, João Luiz Dornelles Bastos, Albert Navarro, David Alejandro Gonzalez-Chica, Antonio Fernando Boing

**Affiliations:** I Universidade Federal de Santa Catarina. Centro de Ciências Rurais. Curitibanos, SC, Brasil; II Universidade Federal de Santa Catarina. Programa de Pós-Graduação em Saúde Coletiva. Departamento de Saúde Pública. Florianópolis, SC, Brasil; III Universitat Autónoma de Barcelona. Facultat de Medicina. Unitat de Bioestadística. Barcelona, España; IV The University of Adelaide School of Population Health. Adelaide, Australia

**Keywords:** Adult, Obesity, epidemiology, Risk Factors, Socioeconomic Factors, Cross-Sectional Studies, Adulto, Obesidade, epidemiologia, Fatores de Risco, Fatores Socioeconômicos, Estudos Transversais

## Abstract

**OBJECTIVE:**

To test whether there is an association between socioeconomic status in childhood and measures of body mass index, waist circumference and the presence of overall and abdominal obesity in adult life.

**METHODS:**

A cross-sectional analysis of a population-based cohort study, including a sample of adults (22–63 years old) living in Florianópolis, Southern Brazil. The socioeconomic status in childhood was analyzed through the education level of the participant’s parents. Height, weight and waist circumference were measured by previously trained interviewers. Linear and logistic regressions with adjustment for confounding factors and stratification of data according to gender were used.

**RESULTS:**

Of the 1,222 adults evaluated, 20.4% (95%CI 18.1–22.8) presented overall obesity and 24.8% (95%CI 22.4–27.4), abdominal obesity. The body mass index and waist circumference averages among women were, respectively, 1.2 kg/m^2^ (95%CI -2.3– -0.04) and 2.8 cm (95%CI -5.3– -0.2) lower among those with higher socioeconomic status in childhood. Among men, waist circumference was 3.9 cm (95%CI 1.0–6.8) higher in individuals with higher socioeconomic status in childhood. Regarding obesity, women of higher socioeconomic status in childhood had lower odds of abdominal obesity (OR = 0.56, 95%CI 0.34–0.90), and no such association was observed among men.

**CONCLUSIONS:**

The socioeconomic status in childhood influences body mass index, waist circumference and obesity in adults, with a difference in the direction of association according to gender. The higher socioeconomic status among men and the lower socioeconomic status among women were associated with higher adiposity indicators.

## RESUMO

**OBJETIVO:**

Testar se há associação entre posição socioeconômica da infância e medidas de índice de massa corporal, circunferência da cintura e presença de obesidade geral e abdominal na vida adulta.

**MÉTODOS:**

Análise transversal em estudo de coorte de base populacional, incluindo amostra de adultos (22–63 anos) residentes em Florianópolis, Sul do Brasil. A posição socioeconômica da infância foi analisada por meio da escolaridade dos pais e mães dos participantes. Estatura, peso e circunferência da cintura foram aferidos por entrevistadores previamente treinados. Foram empregadas regressões linear e logística com ajuste para fatores de confusão, e estratificação dos dados segundo sexo.

**RESULTADOS:**

Dos 1.222 adultos avaliados, 20,4% (IC95% 18,1–22,8) apresentaram obesidade geral e 24,8% (IC95% 22,4–27,4), obesidade abdominal. As médias de índice de massa corporal e circunferência da cintura entre mulheres foram, respectivamente, 1,2 kg/m^2^ (IC95% -2,3– -0,04) e 2,8 cm (IC95% -5,3– -0,2) menores entre aquelas de maior posição socioeconômica na infância. Entre os homens, a circunferência da cintura foi 3,9 cm (IC95% 1,0–6,8) maior nos indivíduos de maior posição socioeconômica na infância. Em relação à obesidade, mulheres de maior posição socioeconômica na infância tiveram menores chances de obesidade abdominal (OR = 0,56; IC95% 0,34–0,90), não tendo sido observada tal associação entre os homens.

**CONCLUSÕES:**

A posição socioeconômica na infância tem influência sobre índice de massa corporal, circunferência da cintura e obesidade em adultos, com diferença na direção da associação conforme o sexo. A maior posição socioeconômica entre os homens e a menor posição socioeconômica entre as mulheres estiveram associadas aos maiores indicadores de adiposidade.

## INTRODUCTION

Obesity is considered a public health problem because of its high prevalence, the significant negative impacts that it produces both on an individual and a collective level, including its linkage to other health problems, such as orthopedic problems, sleep apnea and greater risks for chronic and cardiovascular diseases^26^. The prevalence of obesity is high and increasing in Brazil, with a fourfold increase in males and twofold in females between 1974/1975 and 2008/2009, from 2.8% to 12.5% in males and from 8.0% to 16.9% in females^11^. At the international level, this places Brazil among the 10 countries with the highest prevalence of overweight people in the world, and third among countries with the highest increase in the number of obese people from 1980 to 2008 (20 million), behind only the United States (56 million) and China (42 million)^23^.

Studies indicate that socioeconomic status is strongly associated with the occurrence of obesity^1^ ^,^ ^7^ ^,^ ^13^. However, most studies that evaluate this relationship are restricted to the measurement of socioeconomic status (SS) in adult life, although some investigations have also shown the important role of adverse socioeconomic conditions in the intrauterine and early life periods on the nutritional status of adults^6^ ^,^ ^8^. It is postulated that exposure to adverse socioeconomic conditions in the first years of life influences body composition in the later stages of the life cycle. Childhood is considered a critical period of development, in which the type of dietary intake, environmental characteristics and the presence of infections may affect future growth and body weight^4^ ^,^ ^18^ ^,^ ^21^.

The results of associations between SS in childhood and obesity in adults are mostly from studies in high-income countries^4^ ^,^ ^8^ ^,^ ^21^. Among the few studies conducted in Brazil^1^ ^,^ ^6^ ^,^ ^7^ ^,^ ^9^ ^,^ ^22^, the results are not consistent and differ between the gender and according to the diagnostic criterion of obesity. Particularly among women, the associations are more consistent, with the vast majority of results suggesting a higher frequency of obesity among those with lower SS in childhood. Among men, however, the international literature does not seem to have a consensus in associations; and the few studies performed in two cohorts in Brazil (Pelotas and Ribeirão Preto), indicate that better SS at the beginning of the life cycle is associated with greater occurrence of obesity in adulthood^1^ ^,^ ^6^ ^,^ ^7^ ^,^ ^9^ ^,^ ^22^.

In addition to the effect of childhood socioeconomic aspects on obesity in adults not being clear in middle- and low-income countries, there is no consensus as to the best way to prevent this disease from a population perspective^2^. In this sense, the identification of early factors that influence the epidemiological profile of obesity has the potential to subsidize the development of more efficient strategies to address this problem, either in the population or in certain subgroups. This study aimed to test whether SS in childhood is associated with measures of body mass index (BMI) and waist circumference (WC), and with the presence of overall and abdominal obesity in adult life.

## METHODS

A cross-sectional analysis of data from the EpiFloripa Adult cohort study was carried out, with a baseline that included a representative sample of adults (20–59 years of age), living in the urban area of Florianópolis, capital of the state of Santa Catarina, Brazil, in 2009.In the first stage of data collection, the study population included 1,720 adults. Details on sampling, study population and other methodological aspects are found in a previously published article^3^.

To calculate the baseline sample size of the study, we considered a finite population of 249,530 individuals, a 95% confidence level, prevalence for unknown endpoints of 50%, sampling error of four percentage points, design effect of 2.0, in addition to 10% for correction of absence of response and 20% for control of confounding factors. Based on the calculations, the minimum number of interviews was 1,613, considering a response rate of 80% and coefficient of variation of no more than 11% for prevalence estimates^3^.

The sampling process was carried out in two stages. The primary sampling unit was composed of the census tracts of Florianópolis, with stratification according to the average income of the family head. In the second stage, households were used as units. In 2009, all adults aged 20–59 years old residing in the selected households were eligible to enter the study. Adults institutionalized or physically or mentally disabled to answer the questionnaire were excluded from the survey.

The present study used data from participants from the first follow-up of the cohort, conducted in 2012, in which the participants’ ages ranged from 22 to 63 years old. This data collection was performed through individual face to face interviews conducted at the households of individuals interviewed in 2009. Denials or losses were considered for adults who refused to participate or who were not located by the interviewers after at least four telephone attempts to schedule and four other home visits (at least one at night and one during the weekend).

The main exposure variable was childhood SS, measured by means of parental education level and obtained by applying two questions: “Did your father go to school?” and “What grade/year did your father finish at school?”. The mother’s education level was obtained using the same questions. Information on education level was recorded in years of study completed successfully. It should be noted that, in this study, data from the first cohort follow-up were used because the exposure variable was collected only in 2012.

Due to the breadth of age among the cohort members and the increase in school enrollment of the most recent cohorts (with older individuals presenting parents with fewer years of schooling) (Table 1), the variable parents’ education level was constructed considering the age of the participant. For example, for the older participants (52–63 years old), the median years of study of the parents used in the creation of the SS variable was four years, while for the youngest (22–31 years old), it was 11 years. Childhood SS was then defined as: 1) low, when the parents’ education level was less than or equal to the median and 2) high, when the parents’ education level was higher than the median. Both parent’s education level was used, included separately in the statistics.

**Table 1:** Median years of study of the parents and the participants themselves according to the age of the participant. Florianópolis, state of Santa Catarina, 2012.

Interviewee’s age	Interviewee’s education level	Father’s education level	Mother’s education level
		
	(years of study and p25–p75)	(years of study and p25–p75)	(years of study and p25–p75)
22–31 years	11 (11–15)	11 (5–15)	11 (5–14)
32–41 years	11 (10–16)	8 (4–13)	8 (4–11)
42–51 years	11 (8–15)	4 (4–11)	4.5 (4–11)
52–63 years	11 (7–15)	4 (4–11)	4 (4–11)

The other economic and sociodemographic variables (participant’s gender, age, and current education level) were used in this study to adjust the analyzes and were also collected through a questionnaire applied to the respondents. The gender of the participant was recorded as male or female. The age was calculated from the difference between the date of birth and the date of the interview, being later categorized into 10-year groups. The participant’s education level was recorded in complete years of successful study and was later divided into four categories (0 to 4, 5 to 8, 9 to 11, and 12 or more years of study).

The outcomes were BMI and WC assessed as continuous variables and the prevalence of overall and abdominal obesity. Overall obesity was defined using the values of weight and height. For abdominal obesity, the WC values were used. Data on body weight and WC were collected at the baseline and first follow-up, and height was collected at baseline only. For the present study, the data on body weight and WC of 2012 and stature data collected in 2009 were used. The body weight was measured by a digital scale (GAMA Italy Professional®, model HCM 5110M, with 100 grams resolution and capacity for 150 kg, calibrated before the beginning of the research) with the interviewees barefoot and wearing light clothing, according to the standard procedure described in the literature^25^. The height was measured by a portable stadiometer with a maximum capacity of 200 cm and a graduation of 1 mm, following a standard procedure for its determination^15^. Waist circumference was measured with an inextensible anthropometric tape (Sanny, 200 cm maximum capacity and graduation of 1 mm) with the individual in an upright position. The measurement was taken in the narrowest part of the trunk below the last rib. For individuals without a visible waist, the perimeter was measured at the midpoint between the iliac crest and the last rib. The measurement was performed at the time of expiration^15^. Individuals who were unable to stand up, pregnant women and those who had given birth in the six months prior to the survey were excluded.

The nutritional diagnosis of overall obesity was defined according to the World Health Organization criteria^25^ for BMI values ≥ 30 kg/m^2^, using the same cutoff point for both genders. Abdominal obesity was defined according to gender, according to the WC values, classified according to cut-off points: obesity in men when ≥ 102 cm, and obesity in women: ≥ 88 cm^25^.

The analyses were performed in the statistical program Stata, version 13.0. BMI and WC are described by means and standard deviations and overall and abdominal obesity were presented by percentages and respective 95% confidence intervals. The multivariable analysis of the data was done through linear and logistic regression and the results were considered statistically significant when they presented p-value < 0.05. In total, three models were constructed: the first with the gross analysis of the relationship between childhood SS and obesity markers in adult life; the second, adjusted for the age of the participant; and the third including, in addition to age, current education level in adjustment. Interaction analyses were performed with the gender variable as a possible modifier of effect in the associations (when p < 0.05). Subsequently, in all analyses, the data were stratified according to gender. All estimates were adjusted for the sample weights, considering the effect of the sample design (2009) and the probability of locating the participants in the follow-up of the cohort (2012).

The research was approved by the Human Research Ethics Committee of the Universidade Federal de Santa Catarina (Opinion 351/08 and 1772/11) and all participants signed a free and informed consent form.

## RESULTS

In 2012, 1,222 people aged 22–63 years old were evaluated in the first follow-up of the cohort (71.1% of the baseline), of which 57.3% were women. In addition, the median of current schooling was 11 years (p25–p75 = 9–15). Comparing participants from the study’s baseline to those from the first cohort follow-up, there was a greater loss of men and younger individuals, but no differences were found in the two samples in relation to schooling and nutritional status (Table 2).

**Table 2:** Comparison of characteristics of the participants in the baseline and in the first follow-up of the EpiFloripa Adult cohort, stratified by gender. Florianópolis, state of Santa Catarina, Brazil.

Variable	Men		Women	
	
	Baseline (2009)	First follow-up* (2012)	Baseline (2009)	First follow-up (2012)
			
	n = 761	n = 522	n = 959	n = 700
	%	%	%	%
Age (years)				
20–29	34.2	28,9*	29.2	25,3*
30–39	22.6	22.2	22.9	23.0
40–49	23.8	26.1	26.8	29.9
50 or older	19.4	22.8	21.1	21.9
Education (years)				
0–4	9.1	8.3	9.3	9.3
5–8	14.3	12.9	15.1	15.3
9–11	34.7	34.4	31.8	30.8
12 or more	41.9	44.4	43.7	44.6
Nutritional status (BMI)				
Low weight	1.1	1.0	2.8	3.0
Eutrophy	46.1	45.1	53.4	51.6
Overweight	37.5	38.4	27.0	26.6
Obesity	15.3	15.5	16.8	18.8

BMI: body mass index

* p < 0.05 in the comparison between localized and non-localized.

The average BMI and WC, as well as the prevalence of overall and abdominal obesity in men and women, are presented in Table 3. Differences in the sample according to the prevalence of overall and abdominal obesity were observed in relation to age and current schooling, and the highest percentage of obesity was detected in the categories of older age and fewer years of study (Table 3).

**Table 3:** Distribution of the sample according to the mean body mass index (BMI) and waist circumference (WC) and prevalence of overall and abdominal obesity according to the characteristics of the participants. Florianópolis, state of Santa Catarina, 2012.

Exposure variable (N)	Women (n = 700)				Men (n = 522)			
	
	BMI	WC	Overall obesity	Abdominal obesity	BMI	WC	Overall obesity	Abdominal obesity
							
	Average (SD)	Average (SD)	% (95%CI)	% (95%CI)	Average (SD)	Average (SD)	% (95%CI)	% (95%CI)
Total	26.4 (5.2)	83.3 (13.4)	21.2 (18.2–24.5)	29.7 (26.3–33.3)	26.6 (4.2)	92.3 (12.6)	19.3 (16.0–23.1)	18.8 (15.6–22.5)
Father’s education level								
Low (n = 598)	26.7 (5.2)	83.8 (13.8)	23.3 (19.0–28.2)	32.0 (27.2–37.2)	26.2 (4.4)	90.3 (12.3)	17.7 (13.2–23.2)	16.9 (12.6–22.2)
High (n = 428)	25.3 (4.7)	80.5 (11.9)	12.8 (9.0–18.0)	19.5 (14.8–25.2)	27.0 (4.0)	95.0 (12.8)	21.7 (16.2–28.3)	21.5 (16.1–28.0)
Mother’s education level								
Low (n = 605)	26.8 (5.3)	83.2 (13.0)	23.0 (18.8–27.9)	31.1 (26.3–36.2)	26.2 (4.4)	90.3 (12.5)	17.9 (13.5–23.4)	14.6 (10.6–19.7)
High (n = 466)	25.5 (4.7)	81.8 (12.9)	16.3 (12.0–21.6)	24.3 (19.3–30.1)	26.8 (4.2)	94.0 (13.1)	19.3 (14.3–25.4)	21.7 (16.5–27.9)
Participant’s age (years)								
22 to 31 (n = 293)	24.2 (4.7)	76.3 (9.9)	10.9 (6.7–17.5)	13.0 (8.4–19.6)	25.5 (4.2)	86.9 (11.3)	15.4 (10.0–23.1)	10.5 (6.1–17.3)
32 to 41 (n = 279)	26.5 (5.6)	82.2 (13.9)	23.2 (16.9–31.0)	26.2 (19.7–33.9)	26.9 (4.8)	91.9 (13.2)	19.5 (13.1–27.9)	16.9 (11.1–24.9)
42 to 51 (n = 328)	26.7 (4.6)	84.6 (13.0)	21.3 (16.0–27.8)	31.9 (25.9–39.0)	26.9 (3.9)	93.3 (9.4)	19.1 (13.0–27.0)	19.0 (13.0–27.0)
52 to 63 (n = 165)	27.7 (5.1)	88.2 (13.4)	27.0 (20.9–34.0)	43.3 (36.2–50.7)	27.0 (3.9)	96.6 (13.9)	23.2 (16.5–31.5)	26.7 (19.8–35.0)
Current education (years)								
0 a 4 (n = 165)	28.5 (5.3)	89.1 (11.8)	35.9 (24.9–48.7)	50.0 (37.7–62.3)	26.5 (4.8)	94.1 (15.8)	25.6 (14.0–42.2)	22.5 (11.8–38.7)
5 to 8 (n = 174)	27.8 (5.7)	86.6 (14.8)	32.0 (23.5–41.9)	37.2 (28.3–47.2)	26.5 (4.2)	90.5 (11.5)	20.6 (12.2–32.7)	17.5 (9.8–29.2)
9 to 11 (n = 394)	26.5 (4.9)	84.6 (14.2)	20.6 (15.4–26.9)	32.5 (26.4–39.3)	26.6 (4.3)	91.8 (12.4)	16.7 (11.7–23.1)	15.5 (10.8–21.7)
12 or more (n = 543)	25.5 (4.9)	79.9 (11.6)	14.5 (10.9–19.2)	20.7 (16.5–25.8)	26.6 (4.2)	92.7 (12.3)	20.0 (15.1–25.9)	20.0 (15.2–25.8)

Terms of interaction with the gender variable were verified in the associations of BMI, WC, overall and abdominal obesity with paternal education level, and BMI, WC and abdominal obesity with the variable maternal education level, therefore, the results were presented separately for men and women.

Table 4 shows that BMI and WC were higher among men with higher SS and among women with lower SS. After adjusting for age, BMI values among women were about 1.6 kg/m^2^ (95%CI -2.7– -0.5) lower, and the WC averages were about 4.4 cm smaller (95%CI -6.9– -1.9) in the daughters of parents with more years of study. In males, the association had an inverse sense, in which the WC was approximately 4 cm larger (95%CI 1,1–6,6) in the individuals whose parents were in the higher education level category. In the third regression model, which included the current education level variable, BMI was 1.2 kg/m^2^ (95%CI -2.3–0.04) and WC was 2.8 cm (95%CI -5.3– -0.2) lower in women, and WC in men remained approximately 4 cm higher (95%CI 1.0–6.8) in the categories of parents with higher education level.

**Table 4:** Gross and adjusted coefficient of body mass index (BMI) and waist circumference (WC) of the sample according to the parents’ education level, stratified by gender. Florianópolis, state of Santa Catarina, 2012.

Exposure variable	BMI			WC		
	
	Gross coefficient (95%CI)	Adjusted coefficient (95%CI)^a^	Adjusted coefficient (95%CI)^b^	Gross coefficient (95%CI)	Adjusted coefficient (95%CI)^a^	Adjusted coefficient (95%CI)^b^
Women						
Father’s education level	p = 0.019	p = 0.004	p = 0.043	p = 0.013	p = 0.001	p = 0.033
Low	Ref	Ref	Ref	Ref	Ref	Ref
High	-1.3 (-2.4– -0.2)	-1.6 (-2.7– -0.5)	-1.2 (-2.3– -0.04)	-3.5 (-6.2– -0.7)	-4.4 (-6.9– -1.9)	-2.8 (-5.3– -0.2)
Mother’s education level	p = 0,012	p = 0.001	p = 0.032	p = 0.304	p = 0.039	p = 0.729
Low	Ref	Ref	Ref	Ref	Ref	Ref
High	-1.3 (-2.4– -0.3)	-1.7 (-2.6– -0.7)	-1.1 (-2.1– -0.1)	-1.4 (-4.0–1.3)	-2.5 (-4.8– -0.1)	-0.4 (-2.7–1.9)
Men						
Father’s education level	p = 0.066	p = 0.140	p = 0.100	p = 0.002	p = 0.007	p = 0.009
Low	Ref	Ref	Ref	Ref	Ref	Ref
High	0.8 (-0.05–1.7)	0.6 (-0.2–1.5)	0.7 (-0.1–1.5)	4.7 (1.8–7.6)	3.9 (1.1–6.6)	3.9 (1.0–6.8)
Mother’s education level	p = 0,111	p = 0.283	p = 0.340	p = 0.011	p = 0.075	p = 0.092
Low	Ref	Ref	Ref	Ref	Ref	Ref
High	0.6 (-0.1–1.4)	0.4 (-0.3–1.2)	0.4 (-0.4–1.2)	3.6 (0.8–6.3)	2.4 (-0.2–5.1)	2.4 (-0.4–5.3)

Ref.: reference

^a^ Adjusted for age group.

^b^ Adjusted for age group and respondent’s current education.

Logistic regression results (Table 5) show the same pattern of association. Among women, higher SS was a protective factor for obesity. In the age-adjusted models, the higher education level of the father was associated with lower odds of overall obesity (OR = 0.51, 95%CI 0.29–0.88) and abdominal obesity (OR = 0.48, 95%CI 0.30–0.76). By including current education level in the model, this difference was only maintained for abdominal obesity (OR = 0.56, 95%CI 0.34–0.90). In males, higher SS increased the chance of overall and abdominal obesity, but the differences between the groups did not have statistical significance.

**Table 5:** The ratio of gross and adjusted odds ratio (OR) of overall and abdominal obesity of the sample according to the education level of the parents of the participants, stratified by gender. Florianópolis, state of Santa Catarina, 2012.

Exposure variable	Overall obesity			Abdominal obesity		
	
	Gross OR (95%CI)	Adjusted OR (95%CI)^a^	Adjusted OR (95%CI)^b^	Gross OR (95%CI)	Adjusted OR (95%CI)^a^	Adjusted OR (95%CI)^b^
Women						
Father’s education level	p = 0.032	p = 0.016	p = 0.086	p = 0.017	p = 0.002	p = 0.018
Low	Ref	Ref	Ref	Ref	Ref	Ref
High	0.56 (0.33–0.95)	0.51 (0.29–0.88)	0.61 (0.35–1.07)	0.55 (0.34–0.89)	0.48 (0.30–0.76)	0.56 (0.34–0.90)
Mother’s education level	p = 0.089	p = 0.038	p = 0.317	p = 0.412	p = 0.024	p = 0.308
Low	Ref	Ref	Ref	Ref	Ref	Ref
High	0.66 (0.41–1.07)	0.60 (0.37–0.97)	0.78 (0.48–1.27)	0.80 (0.48–1.36)	0.61 (0.40–0.94)	0.79 (0.51–1.24)
Men						
Father’s education level	p = 0.454	p = 0.466	p = 0.505	p = 0.310	p = 0.433	p = 0.443
Low	Ref	Ref	Ref	Ref	Ref	Ref
High	1.23 (0.71–2.13)	1.22 (0.70–2.12)	1.23 (0.66–2.27)	1.33 (0.76–2.35)	1.26 (0.70–2.30)	1.28 (0.68–2.41)
Mother’s education level	p = 0.818	p = 0.906	p = 0.938	p = 0.064	p = 0.122	p = 0.161
Low	Ref	Ref	Ref	Ref	Ref	Ref
High	1.06 (0.66–1.70)	1.03 (0.64–1.66)	0.98 (0.53–1.79)	1.64 (0.97–2.77)	1.54 (0.89–2.65)	1.54 (0.84–2.84)

Ref.: reference

^a^ Adjusted for age group.

^b^ Adjusted for age group and respondent’s current education.

## DISCUSSION

The results of this study show differences between the genders. Women with lower SS in childhood had higher mean BMI and WC, while in men the association was the opposite, for which higher WC values were found in those with higher SS in childhood. When analyzing the overall and abdominal obesity outcomes, the results maintained the same pattern of association; however, only for abdominal obesity among women, statistical significance was observed after adjusting for adulthood education level.

Among women, the results are consistent with most studies on the subject, regardless of the place of study and the criterion used to define childhood SS^1^ ^,^ ^7^ ^,^ ^8^. As for men, the literature still points to controversial results in this association. Some studies find lower BMI or WC values in individuals with higher SS in childhood, others show a null association, while others show similar results to the present study, in which the measures analyzed are higher in individuals with higher SS in childhood^8^ ^,^ ^21^. It is worth noting that most of the studies that find direct association were carried out in middle-income countries, such as Brazil and China, which seems to show a difference in the pattern of association according to the income of the country of residence^1^ ^,^ ^7^ ^,^ ^12^. Still in the present research, when analyzing the overall and abdominal obesity outcome, no associations were found in the male gender, which corroborates other studies suggesting associations with obesity among women, but not in men^14^ ^,^ ^20^. However, considering that the WC average was about 4 cm higher in the children of parents with higher education level, the SS does not seem to affect the extreme categories, but has an effect on the mean WC. Several studies, including this research, may not find an association when analyzing obesity due to the statistical treatment (categorization or transformation, for example) given to the variables of the analysis.

Different theoretical lines associate childhood SS with obesity in adult life. One of them is based on the fact that childhood is a critical period of development, in which worse socioeconomic conditions can lead to physiological changes in the long term^4^ ^,^ ^18^. In this sense, studies indicate that exposure to adverse conditions during childhood, such as frequent infections and lower energy and protein intake, promotes a series of mechanisms that save energy received and generate stress, promoting the chronic increase of cortisol levels, increased activity inflammatory and metabolic changes, with consequences for the whole life^16^ ^,^ ^24^.

Another theory postulates that the environment in which one lives at the beginning of life will or will not promote better opportunities and lifestyles. Children with low socioeconomic status have fewer opportunities for sports and less access to physical activity and are less participative in physical activities within schools. This relationship with excessive weight gain in adulthood comes from the fact that, in addition to the regular practice of exercise as a child contributing to lower weight, sedentary individuals tend to maintain such a habit. Individuals whose family history of better SS are the ones who practice most physical activity^17^ ^,^ ^18^. Another factor related to childhood refers to feeding at this stage of life, which may influence future food choices and body weight. Studies have shown that the dietary intake of a diet with more vegetables in adulthood is related to the better SS of childhood^10^ ^,^ ^18^.

In this research, differences between genders were found in the magnitude and direction of association of childhood SS and obesity. This fact can be explained by the implication that the socioeconomic disadvantage has on body weight, with a stronger effect among women^19^, which includes the difference in physical patterns imposed on men and women^13^. It is possible to suppose that there are specific cultural demands, by which the excess of corporal weight is covered in negative connotation especially among women. Such demands can reach mainly women of better SS and who have more resources to care for their physical appearance^13^. In addition, men do less weight control, and potentially end up less influenced by issues related to parental control over healthy eating habits in childhood, which includes following a balanced diet^10^.

Another issue to be discussed is the nutritional transition that Brazil is currently undergoing. In high-income countries, men and women with lower SS are more obese, while in middle- and lower-income countries the opposite is true. In Brazil, data from national surveys conducted from the 1970s to 2008–2009 show that obesity in men has always remained more prevalent among those with higher SS^11^. In women, the profile of obesity has been changing, increasing in recent years among those with lower SS and currently reaching similar prevalences^11^, which also corroborates this difference between the genders in the association of obesity with SS.

Most of the studies on this subject analyze only the socioeconomic characteristics of the father as a form of measurement of childhood SS, and the profession is the most common variable. The use of only paternal characteristics may be a limitation since other aspects of the environment in childhood are not analyzed and could also explain this association^19^. The present study, although analyzing only the education level as a variable of SS, used both parent’s education level, since their influence on the health aspects of the children may be different. The father’s education level is more related to the family income and to the acquisition of goods and products, while the mother’s education level tends to have more influence on the education and, consequently, on the children’s living habits^5^. In this study, the associations varied in magnitude when using the education level of the father and the mother as an exposition, and it is not possible to distinguish which one has the greatest effect on the outcomes, even though the father’s education level is shown to have more effect on the WC.

The cross-sectional design with information about the parents’ education level referred to by the participants themselves is a limitation of the study. Despite possible loss of accuracy, this information is relatively stable over time and the results found are less affected by memory bias. In addition, other studies have also used socioeconomic information from the family based on reports in adult life and presented results consistent with those of surveys that collected these data from a longitudinal perspective^8^. Another limitation of the present study is the lack of data that could be used in the adjusted analyses of the research, referring to the children of the interviewees, such as family income at birth and parity.

As strength points, the study included at baseline a representative sample of adults from Florianópolis, which had a high response rate, and included the sample weights with the probability of location in 2012 to reduce the possibility of follow-up bias. We emphasize that there were no differences between the two samples regarding the education level of the study participants. In addition, there are few studies on this subject in low- and middle-income countries, including Brazil.

In conclusion, SS of parents during childhood influences BMI, WC and diagnosis of obesity in adults, indicating that public policies focused on childhood can be used to prevent obesity in adults. Studies on the effectiveness of policies show that it is necessary to develop interventions that can be incorporated into existing health practices and that are maintained in the long term, and those are more effective than specific actions and actions developed during a short period^2^. In addition, the results of this research indicate that actions directed at population groups that are more prone to obesity since childhood should consider differences related to gender. Most current policies are developed for groups with lower socioeconomic status, and especially for women^2^. Public policies may focus on boys from higher SS and girls from lower SS, who make up the groups most prone to obesity in adult life.
